# The Evolving Purposes of Medical Revalidation in the United Kingdom: A Qualitative Study of Professional and Regulatory Narratives

**DOI:** 10.1097/ACM.0000000000001993

**Published:** 2017-11-07

**Authors:** Abigail Tazzyman, Jane Ferguson, Kieran Walshe, Alan Boyd, John Tredinnick-Rowe, Charlotte Hillier, Samantha Regan De Bere, Julian Archer

**Affiliations:** 1 1**A. Tazzyman** is research associate, Alliance Manchester Business School, University of Manchester, Manchester, England.; 2 2**J. Ferguson** is research associate, Alliance Manchester Business School, University of Manchester, Manchester, England.; 3 3**K. Walshe** is professor of health policy and management, Alliance Manchester Business School, University of Manchester, Manchester, England.; 4 4**A. Boyd** is research fellow, Alliance Manchester Business School, University of Manchester, Manchester, England.; 5 5**J. Tredinnick-Rowe** is research assistant, Plymouth University, Plymouth, England.; 6 6**C. Hillier** was research associate, Alliance Manchester Business School, University of Manchester, Manchester, England.; 7 7**S. Regan De Bere** is lecturer, University of Plymouth, Plymouth, England.; 8 8**J. Archer** is senior clinical lecturer and director, Collaboration for the Advancement in Medical Education Research and Assessment, Plymouth University, Plymouth, England.

## Abstract

**Purpose:**

Previous research found professionalism and regulation to be competing discourses when plans for medical revalidation in the United Kingdom were being developed in 2011. The purpose of this study was to explore how these competing discourses developed and how the perceived purposes of revalidation evolved as the policy was implemented.

**Method:**

Seventy-one interviews with 60 UK policy makers and senior health care leaders were conducted during the development and implementation of revalidation: 31 in 2011, 26 in 2013, and 14 in 2015. Interviewees were selected using purposeful sampling. Across all interviews, questions focused around three areas: individual roles in relation to revalidation; interviewees’ understanding of revalidation, its purpose, and aims; and predictions or experiences of revalidation’s impact. The first two interview sets also included questions about measurement and evaluation of revalidation. Data were analyzed using the constant comparative method to understand changes and continuities.

**Results:**

Two main discourses regarding the purpose of revalidation were present across the implementation period: professionalism and regulation. The nature of the relationship between these two purposes and how they were described changed over time, with the separate discourses converging, and early concerns about actual or potential conflict being replaced by perceptions of coexistence or codependency.

**Conclusions:**

The changing nature of the discourse about revalidation suggests that early concerns about adverse consequences were not borne out as organizations and professionals engaged with implementation and experienced the realities of revalidation in practice. Reconciling professional and regulatory narratives was arguably necessary to the effective implementation of revalidation.

## Abstract 2

Supplemental Digital Content is available in the text.

Medical revalidation was introduced in the United Kingdom in 2012, following more than a decade of protracted debate concerning the appropriate methods for ensuring the quality and safety of medical practice. This debate reflected changing wider societal notions of accountability and of quality improvement in health care.^1–4^ Revalidation requires doctors to demonstrate on a regular basis that they are up-to-date and fit to practice, by bringing a portfolio of evidence (called supporting information) of continuous professional development (CPD), peer and patient feedback, quality improvement or audit, and significant events to their annual appraisal. The revalidation and appraisal framework is based on “Good Medical Practice” (GMP), the core guidance for doctors published by their professional regulator, the General Medical Council (GMC). This consists of four domains of performance: knowledge, skills, and performance; safety and quality; communication, partnership, and teamwork; and maintaining trust. GMP requires that doctors should be “regularly reflecting on [their] standards of practice and the care [they] provide.”^5^ Evidence of this reflection is a key component of revalidation. Every five years a “responsible officer” (RO), a senior doctor employed by each health care organization, makes a revalidation recommendation to the GMC. Doctors who do not engage with revalidation can, ultimately, lose their license to practice. In the United Kingdom the medical licensing system is run by the GMC, which holds two registers of those eligible to work in the UK health service, the Specialist and General Practise (GP) Registers, and doctors must be registered on one or both of these. The medical Royal Colleges in the United Kingdom are responsible for setting standards within their field and for supervising the training of doctors within that specialty. Internationally, revalidation is most similar to New Zealand’s practicing certificate and recertification and the American maintenance of licensure and certification.^6–8^ The implementation of revalidation commenced in December 2012, with the intention that all doctors in the United Kingdom would have undergone revalidation by March 2016.

Revalidation signals a fundamental change in how the medical profession is regulated and is a complex new intervention that has evoked considerable critical debate amongst health care professionals.^4,9,10^ There has been much deliberation and divergence of opinion over the purposes of revalidation and a dichotomy within the debate over whether revalidation should or could serve what might be termed the purposes of regulation or of professionalism.^11–13^ Professionalism has been variously defined but can be explained as a model where the organization of, and control over, work is fulfilled by the profession itself instead of by the market or by some form of administrative hierarchy.^14^ It is underpinned by a progressive, intrinsically motivated ambition to define and drive up quality standards. In this context, it is used to refer to the notion that revalidation should seek to raise professional standards by capitalizing on the intrinsic motivations of doctors as professionals, for example, by increasing involvement in CPD and fostering quality improvement activities. Regulation conversely can be defined as sustained and focused control over activities valued by society, which is exercised by a public agency.^15^ It can be understood as primarily a retrospective summative assessment of performance^16^ and in this context is largely concerned with whether or not doctors meet minimum standards and are safe and fit to practice. It is an extrinsic process, shaped mainly by notions of accountability. Various elements of these two contrasting perspectives are considered in the literature.^2,11,17,18^

Previous research found professionalism and regulation to be competing discourses within the professional and revalidation policy-making community when plans for revalidation were being developed in 2011, but had not yet been introduced.^11^ This diversity of opinion regarding the purpose of revalidation was identified as potentially problematic for the development and successful implementation of the policy.^11^ It was noted, however, that “revalidation policy has always been a product in and over time, and it must be acknowledged that our interviews may represent only one moment in this time continuum.”^11^^(p92)^ In this study, by analyzing key stakeholder interviews at different points in time before and during the implementation of revalidation, we expand on this line of enquiry and explore how these competing discourses have developed, and how the purposes of revalidation have evolved.

## Method

We undertook a qualitative investigation of the initial stages of implementing revalidation in the United Kingdom between 2011 and 2015, from the perspective of those who were responsible for its development and early implementation. We conducted 71 interviews with a purposeful sample of 60 UK policy makers and senior leaders at three different points during the development and implementation of revalidation: 31 in 2011, 26 in 2013, and 14 in 2015. Participants were recruited purposively because of their role organizationally or their involvement in the development of medical revalidation. We approached them by e-mail with no compensation for involvement in the study being given. Interviews typically lasted between 45 and 60 minutes. The first two interview sets were all conducted face-to-face and the third set face-to-face or by telephone, according to participant preferences. The first set of interviews, from 2011, was conducted prior to the introduction of revalidation; the second set in 2013, shortly after the introduction of revalidation, and the final set in 2015, once the process of implementation had been under way for three years. A discourse analysis of the 2011 interviews has previously been presented.^11^ The topic guides across all three interview sets focused around three main areas: individual roles in relation to revalidation; interviewees’ understanding of revalidation, its purpose, and aims; and predictions or experiences of revalidation’s impact (see Supplemental Digital Appendix 1 at http://links.lww.com/ACADMED/A501). The first two interview sets also included a section on the measurement and evaluation of revalidation, as they were part of the development of an evaluative framework for revalidation.

**Chart 1:**
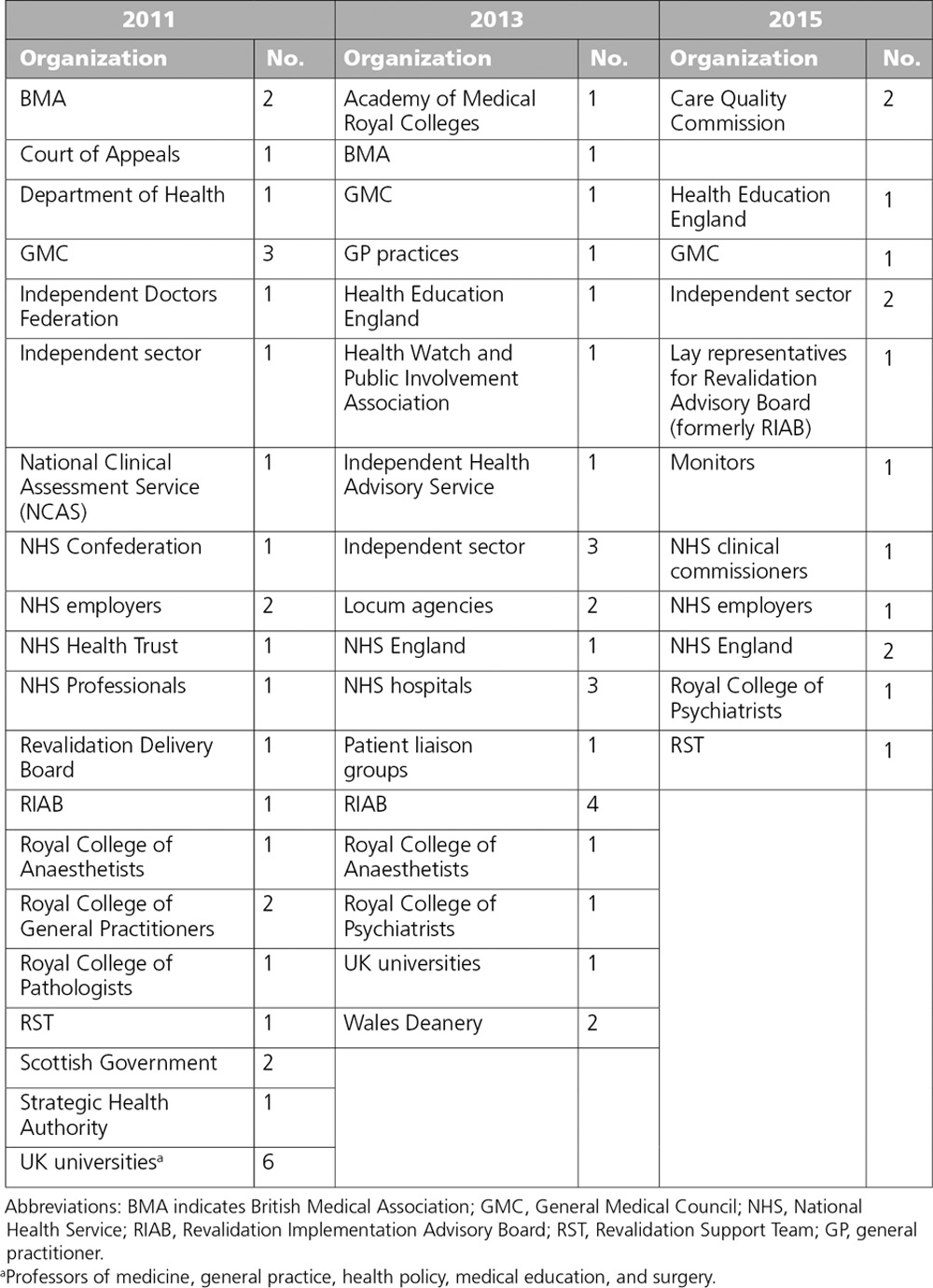
Interviewee Organization Affiliation, From a Study Evaluating the Development of Medical Revalidation in the United Kingdom, 2011–2015

The interviewees were all key actors in the development and design of revalidation, with 11 participants being interviewed at two time points and 49 interviewed once. No participant was interviewed at all three data points because of changes to senior involvement over a five-year period. The participants’ affiliations to organizations are summarized in Chart 1.

To assist the organization of data, we digitally recorded interviews, transcribed them, and then imported them into Dedoose qualitative data analysis software (SocioCultural Research Consultants, Los Angeles, California). Transcripts were encrypted and stored securely, so they did not need anonymizing. Interview transcripts were coded by two members of the research team (A.T. and J.F.) using a directed content analysis approach.^19^ We developed a coding framework based on previous findings from our earlier analysis of the 2011 interview set. It was structured to explore what participants understood to be the purpose of revalidation and how the previously identified discourses of professionalism and regulation featured within their narratives. Following coding, two members of the research team (A.T. and J.F.) analyzed the data across the three interview stages, using the constant comparative method,^20^ to understand changes and continuities over time. We enhanced rigor and trustworthiness of the data analysis through researcher triangulation. Throughout its development, the coding framework was discussed with all the team members, as were the themes and findings that arose from it. A few minor disagreements occurred regarding decisions, and these were resolved through consensus discussion. Initial coding was also verified across two researchers (A.T. and J.F.) to ensure consistency of the framework’s application.

Ethical approval for this study was awarded by the Peninsula College of Medicine & Dentistry research ethics committee (no. 12/13-122), by the Plymouth University Faculty of Health and Human Sciences & PSMD Research Ethics Committee (no. 15/16-486), and by the University of Manchester ethics committee (REC 15028).

## Results

To demonstrate how the key discourses concerning revalidation have evolved over time, we have organized our findings chronologically, and identify both areas of continuity and of change during the period in which revalidation was being introduced and implemented.

### 2011

As was noted earlier, two dominant discourses about the purposes of revalidation were identified in the interviews in 2011, which we termed professionalism and regulation. While these purposes were discussed simultaneously in many participants’ narratives, a perceived conflict or incompatibility between professionalism and regulation was articulated as a concern by the majority of interviewees. This incompatibility or conflict featured in different ways within the 2011 interviews. For some, the two purposes were presented as distinct and in competition:

What it is not, which some of the Colleges have been looking at, is driving up the standards. It is actually about assuring the standard. Driving up standards is professional development and everything else. Revalidation is ensuring that people do not fall below a particular minimum standard level.… We are not looking for excellence in revalidation; we are looking to make sure that people are OK and safe. (2011)

[The government] did want to bring doctors down a peg or so, there was this feeling that they are prima donnas and that they do what they want and so on. I think there were many attempts to try and, for want of a better word, bring them down a peg or two. I think that whilst they saw revalidation as a tool it was a part of the range of things that they did.… That was the approach used: regulation. (2011)

For others, though both purposes were acknowledged, their separateness was articulated and the differences between them were emphasized. For example, some thought the purpose of revalidation had changed, from the identification of poor performance to promoting professionalism and standards of practice. In these narratives, there was a presumption that the two purposes would not or could not coexist:

What we were originally told would be a basic quality assessment that almost every doctor should pass, except the really bad ones who really needed remediation, was being turned round into a scheme that appeared determined to identify those doctors who were paragons of practice, those doctors who exemplified all of the attributes required of a doctor and exemplified them at high level. (2011)

The thing about it is it was always designed to try and recognize another Shipman^*^ early, but it will never do that of course. Things have moved on from that. Now it is designed to raise standards across the board by establishing what is the recognized standard practice and ensuring that everybody fulfills that…. So I think revalidation is designed to try and improve medical practices’ standards across the board but it is not, and it should not be regarded as, just a way of looking for bad apples because that is very difficult. (2011)

Moreover, some interviewees thought that the two purposes were detrimental to each other, with the achievement of one seen as negatively affecting the ability to achieve the other:

By and large I think the public see this as screening excluding those not fit-for-purpose [sic] and the profession sees it as shifting the curve to the right. I could go on and on about this … but essentially the mechanisms you use for one are not necessarily the mechanisms you use for the other. One process has to be formative and the other is summative and one of the dangers of our current position is that we are trying to use the single system to be both formative and summative and that is not easy to do. (2011)

There is a tension…. There is the really supportive element of appraisal and potential revalidation as well as the more objective summative assessment process.… I think we have got a bit of work to do to be able to make clinical physicians confident that if they do genuinely have anxieties or concerns that they have a way of expressing those but that it will not necessarily have a major impact on their initial appraisal outcome until some of these issues have been dealt with. (2011)

### 2013

By 2013, when the implementation of revalidation was under way and many interviewees now had some experience of the process (often both in leading revalidation and in being revalidated themselves), professionalism and regulation still featured as the two dominant purposes of revalidation. But the experience of engaging in revalidation seemed to have altered some interviewees’ understandings of the relationship between the two purposes. Some interviewees still spoke of the two purposes as either distinctly separate or in conflict:

If the system’s not picking out the poorly performing doctors then the system’s not working … one of the primary purposes of the system is to identify poorly performing doctors. (2013)

What I think it will do is it will drive quality up because I think what it requires doctors to do is to reflect on their practice and in so doing it should, at the end of the day, ensure that a doctor is practicing within their competencies and is operating against a quality framework in whatever their specialized area is whether a GP or a specialist. (2013)

The trouble with revalidation is it’s trying to combine two things; in the old days appraisal was more about things like mentoring or supervision where the idea it was a safe environment … now it’s got a big performance management component to it and I’ve never been convinced that you can safely combine those into one. (2013)

However, many more participants began to speak of the two purposes as necessarily coexisting, often with a pragmatic recognition that both were needed in practice. Overall, the fundamental tensions or conflicts between them anticipated by the majority of participants in 2011 seemed not to have materialized in practice. There was now a growing view that these purposes might coexist, and there was some reframing of the earlier dichotomy between dealing with “poor” performance and promoting professionalism.

I do think revalidation is a regulatory mechanism to ensure proper standards [minimum standards] in the practice of medicine. I think it’s a—I think building on what I said earlier it was and can be and should be a means to ensure that people don’t just keep their nose down day to day doing clinical work without reflecting how to improve, without reflecting how to make things better. (2013)

I think even y’know in the last two three years [the profession] have become much more aware of doctors who are not performing prop[erly], not performing well, it’s and trying to develop other doctors with the skills to be able to manage doctors well, and that performance management is going to be quite a challenge but I think it can happen and revalidation is really the springboard for ensuring that that takes place. (2013)

I think it’s really to hold doctors and indeed employers responsible for making sure there are policies and procedures in place to make sure doctors are looking at their own practice and undertaking activities to keep themselves up-to-date and improve their practice it’s um to make sure we’ve all got policies and procedures in place for handling concerns about doctors and mechanisms for capturing feedback … it’s about someone casting their eye over and making a sound judgment it’s about making us all accountable. (2013)

### 2015

The final set of interviews was conducted in 2015 once the implementation of revalidation had been under way for three years, and at a point when almost half of doctors in the United Kingdom had been through their first experience of revalidation. Participants at this stage were far more familiar with how revalidation worked and experienced in the processes it involved. There were still some perceived tensions between the professional and regulatory purposes of revalidation, and the ability of processes like appraisal to serve both purposes:

I think hence doctors who have to get through their revalidation portfolio, and [need] their recommendation to be positive, I think are possibly sanitizing their reflections, and being less reflective, less self-critical than maybe they should be. Or, if the rhetoric of revalidation would be that it’s a place to reflect, they are worried that if their reflection is too critical … they may actually water down their reflection just enough to be engaged, but not too much. (2015)

There is a danger through revalidation that the appraisal gets a bit dumbed down to the basics and it becomes almost a sort of tick-box exercise, have you got 50 credits, two significant events, and have you done an audit in the last five years, patient survey and colleague feedback? (2015)

However, the 2015 interviews in general seemed to show that interviewees had found that these two purposes could be reconciled in practice. Both were frequently cited as dual aims needing to be met for revalidation to be considered a success. Revalidation was often situated as having or requiring a two-pronged approach:

I think a lot of the value in revalidation is in the ability of reflection and a strength of sort of governance system with that to really provide doctors with an opportunity to develop and improve. (2015)

So for NHS [National Health Service] doctors, doctors in large, independent hospitals, they’re probably used to having a management appraisal. For doctors in primary care, they’re probably very used to having an appraisal that is very supportive and developmental. And through revalidation, we’ve now got a system which is a bit of both. So it attempts to make judgments about the quality of the doctor’s practice and is also supportive and developmental. (2015)

The hold to account was more to do with making sure that people meet the requirement of the GMC in terms of good medical practice, but the skills are more about reflection and facilitating appraisees, you know, to recognize that they may well be producing evidence to support good practice but as well as that they need to look to see whether the evidence that they are producing is helpful, whether they need to address any areas. (2015)

Some participants went further than seeing professionalism and regulation as coexisting dual purposes of revalidation, and suggested that they were interconnected, and even somewhat codependent.

So it’s formalized … the whole process that was already happening in much more of an ad hoc way.… That is a good thing because I firmly believe that revalidation has improved the quality and the professionalism of doctors in this country because it’s forced the issue, whereas before it was much more voluntarily. (2015)

The doctor has to demonstrate, to the extent the system demands of him or her, that he is a suitable, if you like, suitable to practice. That’s quite different from a model in which the regulator only deals with those situations in which there’s a strongly suspected view that the doctor isn’t fit to. It moves it from reacting to only when there’s a problem, to proactively trying to stop problems occurring, and broadly, improve standards … it drives them in the right direction across the board. So it’ll drive them in the right direction in terms of people’s capability, their clinical competence, it’ll drive them in the right direction in terms of probity. (2015)

I think the RO regulations more or less force a better quality appraisal conversation because it’s at its heart. It’s a, sort of, performance management system where if we’re appraising one another, as it were, we do have somebody then saying, well, what was the outcome there and we can’t recommend relicensing over the five-year cycle. The Responsible Officer themselves are then under quite noticeable external scrutiny by their own RO and in due course by the General Medical Council accepting or rejecting the recommendations that they make. (2015)

## Discussion

Our analysis of revalidation stakeholder interviews undertaken in 2011, 2012, and 2015 found that, with regard to the purpose of revalidation, two main discourses were present across the implementation period under investigation: professionalism and regulation. However, the nature of the relationship between the two purposes and the way they were described has changed over time, with the separate discourses converging, and early concerns about actual or potential conflict being replaced by perceptions of coexistence or even codependency. Before the implementation of revalidation, many stakeholders articulated fundamental concerns about a conflict between these two purposes, emphasized their differences, and expressed doubts about which might prevail, or whether revalidation could serve both purposes. During the implementation of revalidation, the relationship between these purposes began to change, and it seems that the experience of “doing” revalidation led stakeholders to find that they could at least coexist without too much dissonance in practice. Indeed, some stakeholders began to see the dichotomy between professional and regulatory purposes as somewhat artificial, and to argue that dealing with concerns about poor practice and seeking to improve professional standards were complementary and even codependent purposes.

The introduction of revalidation has been the most significant change in medical regulation in the United Kingdom in many decades, and has involved a fundamental reconfiguration of the relationships between doctors, the organizations in which they work, and the professional regulator, the GMC. Before the implementation of revalidation, there were many calls to define and clarify its purpose.^9,21^ But it seems that, in part because this was a contentious reform, there was considerable (perhaps necessary) ambiguity about its purpose or purposes and the way its implementation might affect the medical profession and its notions of professionalism and accountability. In practice, the concern that revalidation might subvert or undermine professionalism, replacing its intrinsic values and purposes with a set of extrinsic ones imposed by society or government, does not seem to have been realized. Rather, it seems that, for senior medical leaders and revalidation stakeholders, a way has been found to adapt the conceptualization of professionalism and professional regulation to enable the acceptance and incorporation of revalidation.

The process of evolution in the purposes of medical regulation and the nature of medical professionalism that we have observed is far from new, and empirical sociological accounts of how doctors were regulated in the 1990s^22,23^ vividly demonstrate how much has changed over the last three decades. The medical profession has accepted and incorporated a series of innovations in governance, accountability, performance measurement, and regulatory oversight which may seem at odds with traditional ideas of professional self-regulation. These reforms have been accepted and even embraced or advocated by elites and leaders of the profession as a necessary means to enhance professionalism while retaining professional control. The introduction of revalidation has been positioned as the latest step in satisfying public and political expectations of accountability from the profession while also being an act of professionalism in itself, because it demonstrates an intrinsic motivation to improve professional standards and ensure the quality of medical care. From this perspective, a reconfigured notion of professionalism thus assumes that individual doctors acknowledge the need for themselves to be held accountable and accept that revalidation is the means by which this accountability is enacted.

As the first cycle of medical revalidation comes toward completion, the GMC is consulting on how the practical implementation of revalidation might be improved,^24^ but the principle that doctors should be required to demonstrate periodically that they are fit to practice has moved from being a contentious proposal which met with much opposition in the decade before it was introduced, to being largely accepted in principle and in practice.

### Limitations

Our findings are based on qualitative interviews with elite stakeholders and members of the profession involved in the development of revalidation. The position of these individuals may mean that to fulfill their roles, greater buy-in is needed than the wider profession, and so the generalizability of the views presented in this paper may be to a degree limited. This research points to the need for further investigation into the practice of revalidation, with a focus on the experiences and perspectives of those engaged with the policy at different levels of the profession.

### Conclusion

The changing nature of the discourse about revalidation during the period of its implementation suggests that as organizations and professionals engaged with and experienced the realities, early concerns about adverse consequences were not borne out. Moreover, it seems that the effective implementation of revalidation involved a necessary reconciliation of professional and regulatory narratives in ways that have embedded notions of accountability and regulatory oversight in a redefined, modern conceptualization of professionalism.

## Acknowledgments:

The authors would like to thank all the interviewees for taking part.

## Supplementary Material

## Footnotes

* Harold Shipman was a British general practitioner found guilty of murdering 15 of his patients in 2000. In the wake of his conviction, much of Britain’s legal structure concerning health care and medicine was reviewed.
