# Supplementary material for: The Evolving Purposes of Medical Revalidation in the United Kingdom: A Qualitative Study of Professional and Regulatory Narratives
Source: Acad Med. 2017 Nov 7;93(4):642–7. doi: 10.1097/ACM.0000000000001993 (PMC5895134; doi:10.1097/ACM.0000000000001993)
Supplement: Supplementary file 1 [file acm-93-642-s001.pdf]

Supplemental Digital Appendix 1

Interview Schedule Guide, From a Qualitative Study of the Purposes of Medical Revalidation in the United Kingdom, 2011–2015

Interview schedule design

Aims

1. Elicit new theories of how revalidation influences medical performance, particularly through its impacts on organisational systems and processes related to medical performance (job planning, CPD, clinical audit)
2. Confirm/further develop theories previously derived from the research/evaluation literature, policy analysis and previous interviews
3. Snowballing: signposting to additional information sources (e.g., project evaluations, additional interviewees)
4. Secure appropriate further engagement in our project (e.g., input to prioritise theories for further investigation; membership of project steering group)

Key considerations

1. Time constraints – there is a lot of ground to cover (revalidation has a number of components and there are many organisational systems and processes; apart from appraisal, and perhaps the organisational systems and processes explicitly covered in appraisal (e.g., audit, complaints, CPD), there has been little attention paid to theories of change, so interviewees may struggle to articulate such theories, taking up), and interviewees may not give us a lot of time. We also need to get on with the interviews so that they can inform WP2.
2. The place of theory confirmation/development in the interviews - uncertainty/tensions among research team members about: how to identify/construct theories from the literature, policy analysis and previous interviewees; the validity of the research team constructing theories (albeit based on data) and then checking them out with interviewees – would this be akin to asking leading questions? On the other hand, without some structuring of the interviews, there is a danger that they will not go deep enough to produce additional information, but will merely provide “surface” explanations.
3. The nature of the theories (context, mechanism and outcome triads?) to be elicited. Mechanisms as used in realist evaluation can be hard to grasp. One accessible way of thinking about them is in terms of “resources and reasoning” that actors may make use of/apply. This has been further complicated by our use of the term “mechanism” to refer to organisational systems and processes related to medical performance.

Suggested way forward

1. Regard each individual interview as contributing some complementary pieces of an overall jigsaw, rather than trying to cover all of the ground in every interview. This would mean Covering a few areas in depth/detail in each interview:
    a. Areas not well-covered by previous interviews conducted during WP1. This can be coordinated through:
        i. Weekly team discussion of priorities and emerging learning
        ii. 24 hour turnaround on transcription for the first few interviews
    b. Areas that the interviewee is knowledgeable about and/or interested in – so that the interview is useful to both sides
2. Focus on new theory elicitation rather than on theory confirmation, at least in initial interviews.
    a. This will give the research team more time to consider how theory confirmation/development and triangulation will be integrated into the project, and we may gain useful learning from the first few interviews that will help with this. In view of the difficulty/complexity, better to keep it simple for the time being.
    b. If we can set up a means of evaluating/prioritising theories for further investigation/testing through the survey, case studies and further literature evidence review (E.g. the steering group plus interviewees who are interested to participate), then this can provide a theory confirmation/validation/development function. We would need to schedule this in the light of interview dates arranged and the survey etc. dates.
3. Provide some structure for the interview, but at a fairly high level.
    a. Corresponding to 3 perspectives/dimensions - key areas of: medical performance; organisational systems and processes for managing medical performance; RO revalidation components. This will facilitate monitoring of what ground the interviews cover (see above), will help to stimulate new thinking by the interviewee by bringing in these 3 perspectives, but will not overly lead/prompt them.
    b. Depth can be obtained through follow-up questioning within each area that is addressed, based on consideration of generic concepts from realist evaluation: context, mechanism, outcome, resources and reasoning. This will be elaborated further if agreed as a way forward. It would have the additional benefit of helping to clarify what we mean by "mechanism," etc., so that research team members gain a common understanding.

4. Priority for theory construction - identify/construct generic/common theories that relate to appraisal and the sources of information relating to organisational processes and systems that are to be considered within appraisals (e.g., CPD, audit, complaints etc.). If there are various common theories that could apply to all of these (as would appear to be the case from the way these aspects are incorporated into appraisal guidance as just a list of items), then we can streamline the investigation of these areas in the interviews by referring to/building on these common theories – whether an interviewee is considering CPD or audit, their theories can be compared with these common theories. To illustrate, some possible common theories might be:
    a. Coverage/Compulsion: Revalidation requires information to be presented at appraisal regarding CPD/audit etc. undertaken; this will compel *all* doctors to participate in these activities [Some issues: token VS genuine participation? Capacity of organisational systems to accommodate greater doctor participation? Etc.]
    b. Rigour/robustness VS Ritual/formalism: Because successful revalidation is important to doctors and their employers, appraisal will be of a higher quality, and this will improve the quality of doctor engagement in CPD, audit, etc. [Some issues: could still be a tick box exercise? Lack of appraiser skills, time? Etc.]

digital content for Tazzyman A, Ferguson J, Walshe K, Boyd A, Tredinnick-Rowe J, Hillier C,
e S, Archer J. The evolving purposes of medical revalidation in the United Kingdom: A
dy of professional and regulatory narratives. Acad Med.

Manchester Business School, University of Manchester

Evaluating the development of medical revalidation in England
INTERVIEW GUIDE – STAKEHOLDER INTERVIEWS, WORK PACKAGE 1

Introduction [2 mins]

- Purpose of interview – to consider the impacts revalidation might have on other activities related to medical performance (job planning, CPD, clinical audit, etc.), and why, including facilitators and barriers. The focus is more on rationales underlying the policy design than on evidence of impact – we will be collecting evidence systematically later in the project. We are interviewing about 20 people who have been involved in the development or implementation of the revalidation model.
- How much time do you have for this interview?

Informed consent [5 mins]

- Have you received and understood our project information sheet? Any questions?
- Run through consent form, checking/recording consent. Any questions?
- Switch on tape, if appropriate.

Background and involvement in revalidation [3 mins]

1. What is your role at [Organisation name]?
2. Please describe your involvement in the development and implementation of revalidation.

Impact [40 mins]

For each of the following 3 perspectives on revalidation (Dr outcomes; Dr performance systems; RO responsibilities):

a. Identify key areas to focus on (prior to the interview, reorder the focusing prompts as appropriate, so that gaps are addressed first; some areas may be omitted).
b. Explore each area by asking generic questions:
    a. How do you think revalidation might produce those impacts (how do you think the advent of revalidation might change the thinking and behaviours of different actors: Dr’s, ROs, managers, etc.)?
    b. Do you think impacts might vary? What factors do you think might affect impact?

i. Organisation-level factors (e.g., sector/type; low/high performer; culture, etc.)
ii. Individual-level factors of ROs, Appraisers or Doctors (e.g. low/high performers; stage of career; specialism, etc.)?

c. What unintended consequences do you think there might be?

1. Dr outcomes: What impacts do you think the implementation of revalidation policy might have on doctors?
   Focusing – what about:
   a. *Health and well-being (e.g., stress and burnout)?*
   b. *Clinical expertise?*
   c. *Behaviours and attitudes (e.g., motivation, values; reflexivity, insight)?*
   d. *Education (in qualifying; ongoing professional development)?*
2. Performance systems: What impacts do you think the implementation of revalidation policy might have on organisational systems and processes related to managing medical performance?
   Focusing – what about:
   a. *Appraisal and personal development planning (PDP):*
      i. *declaring acceptance of obligations in Good Medical Practice prior to appraisal (e.g., on probity, personal health);*
      ii. *presenting appropriate supporting evidence- on CPD; quality improvement activity; significant events; feedback from colleagues; feedback from patients; review of complaints and compliments;*
      iii. *demonstrate progress against PDP; agree a new PDP and associated actions*
      iv. *Confirmation in the appraisal summary of the appraiser's duties*
      v. *Provision of appraisal records to the responsible officer*
   b. *Individual Performance Review [should be separate to appraisal]*
   c. *Job planning*
   d. *Continuing professional development (In-service training; continuing medical education)*
   e. *Clinical Audit (audit and feedback; significant event analysis; self-audit); quality improvement*
   f. *Complaints management*
   g. *Incident reporting (others errors; one's own errors); incident investigations; voicing concerns about patient safety; whistleblowing*
   h. *Risk management and medical negligence litigation*

   i. *Disciplinary and conduct procedures*
   j. *Occupational health referral/support*

3. What impacts do you think the RO/DB responsibilities with regard to revalidation might have? *Focusing – what about:*
   a. *Making recommendations to GMC about doctors' fitness to practice*
   b. *Ensuring designated body carries out regular appraisals*
   c. *Establishing and implementing procedures to investigate concerns about fitness to practice [additional requirements in England only – e.g., ensuring appropriate measures are taken to address concerns – re/training, rehabilitiation, work experience, etc.][also referring concerns to GMC; monitoring compliance with GMC conditions or undertakings of a Dr;]*
   d. *Maintain records of Drs' fitness to practise evaluations, including appraisals and any other investigations or assessments*
   e. *Maintaining assurance of the medical appraisal process, including assurance review of medical appraisers*
   f. *RO [England only] – ensure appropriate checks when Dr's contract to be employed /supply services*
   g. *RO [England only] – review general performance information, identify issues and ensure DB addresses them*
   h. *DB providing RO with sufficient funds and other resources necessary to discharge their responsibilities [includes obtaining funds from certain Dr's E.g., locum agency]*

Reflections [5 mins]

1. Is there anything in the revalidation model that you think should be different, or you have concerns about?
2. To what extent do you think impacts will be sustained over time? Why? What is [Organisation name]'s role/responsibility for securing change/improvement in revalidation practices?

Additional information [5 mins]

1. Are there any issues we haven't covered that you would like to talk about?
2. Are there any questions you would like to ask me?
3. Is there anyone who you think would be particularly interesting for us to interview?
4. Can you signpost us to any evaluations or projects relating to revalidation that might provide information about its impact?

5. What further contact with this research project would you be interested in, if any?
    a. Receive notification of publication of key reports, articles, presentations etc.
    b. Join project steering group [6 monthly meetings]
    c. Help us prioritise issues to investigate further in our RO surveys and case studies:
        i. By email?
        ii. By attending a workshop?
    d. Other (please state)
6. What happens next – transcribe interviews; identify themes; prioritise issues for investigation
7. Thank you
